# The prevalence and medical costs of neovascular glaucoma in a tertiary medical center in China from 2016 to 2024

**DOI:** 10.3389/fpubh.2026.1859242

**Published:** 2026-06-25

**Authors:** Qianqian Ji, Fangyi Zhang, Xinyi Zhao, Kang Feng, Mengyao Zhang, Ying Hong

**Affiliations:** 1 Department of Ophthalmology, Peking University Third Hospital, Beijing, China; 2 Beijing Key Laboratory of Restoration of Damaged Ocular nerve, Peking University Third Hospital, Beijing, China

**Keywords:** age, China, etiology, gender, medical cost, medical visit, neovascular glaucoma

## Abstract

**Introduction:**

Neovascular glaucoma (NVG) is a severe, vision-threatening secondary glaucoma. With a higher incidence and earlier onset of its primary diseases, NVG may increase the healthcare burden. This study investigate the demographic characteristics and medical costs of NVG in a tertiary medical center in China.

**Methods:**

This retrospective cohort study included patients diagnosed with NVG, angle neovascularization, or rubeosis iridis at Peking University Third Hospital from January 2016 to December 2024. Data collected included demographics (gender, age, diagnosis, and etiology), treatment (conservative or surgical treatment), number of visits, and medical costs.

**Results:**

A total of 1,275 patients were included, with a mean age of 59.9 ± 16.3 years (range 2–98). 64.5% of patients were aged 45 - 75 years. Male patients predominated (828 vs. 447, ratio 1.9:1) and had an earlier age of onset than females (56.6 ± 16.1 years vs. 62.3 ± 14.8 years; *p* < 0.001). Diabetic retinopathy (DR) was the most common cause (758 cases, 59.5%), followed by retinal vein occlusion (186 cases, 14.6%). Treatments included conservative (819 cases) and surgical (457 cases) approaches. Patients used an average of 2.4 ± 0.4 antiglaucoma medications (range 2–6). Surgical interventions included vitrectomy and antiglaucoma surgery. The number of visits ranged from 1 to 210 times, with a median of 2 (IQR 1–7). 1053 patients (82.5%) had 1–10 visits. Median treatment duration was 49.8 days (IQR 10.7–190.0). The highest per-outpatient costs were in the 45–75 age group (400.1 ± 376.1 yuan), while the highest per-surgical costs were in the 18–45 age group (9379.3 ± 10543.6 yuan).

**Discussion:**

NVG requires increased attention to the risk of DR in middle-aged and older men. Developing age-specific strategies for allocating outpatient and surgical resources is essential to reduce the economic burden of NVG across different age groups.

## 1 Introduction

Neovascular glaucoma (NVG) is a severe and refractory secondary glaucoma, resulting from retinal or anterior segment ischemia. NVG can be divided into three clinical stages: rubeosis iridis, open-angle glaucoma, and angle-closure glaucoma (1, 2).

Studies have reported that the main cause of NVG is retinal ischemic diseases, including diabetic retinopathy (DR) and retinal vascular occlusion (1). Treatments involve panretinal photocoagulation (PRP), anti-vascular endothelial growth factor (VEGF) injections, and surgical interventions such as filtering surgery, glaucoma drainage implantation (GDI), and cyclophotocoagulation (CPC). Many patients require combined therapies (3–7).

With the increasing incidence of systemic diseases like diabetes and hypertension in younger populations, NVG may present with earlier onset, while rising life expectancy may contribute to a higher incidence of NVG (8–12). To reduce socioeconomic burden and improve patients' quality of life, early NVG detection and identification of factors influencing medical costs are needed. Although combined population-based screening for multiple blindness-causing eye diseases has proven highly cost-effective in China (13), its role in NVG prevention remains unclear. In glaucoma more broadly, medical costs increase with disease severity but decrease annually over the treatment course (14). Some studies compared the cost differences between conservative and surgical treatments and suggested trabeculectomy was more cost-effective than medical treatment (15, 16). However, health economic studies specifically on NVG are still limited.

The aim of the study was to analyze the demographic distribution and medical costs of NVG patients at Peking University Third Hospital between 2016 and 2024.

## 2 Methods

This was a retrospective cohort study. All patients diagnosed with NVG, angle neovascularization, or rubeosis iridis at Peking University Third Hospital between January 2016 and December 2024 were identified from the Hospital Information System (HIS). Patients were identified by the Chinese Clinical Modification of ICD-10 (ICD-10-CCM) codes (H21.101, H21.102, H40.501) and clinical terms in the medical records, including iris neovascularization, rubeosis iridis, NVG, and hemorrhagic glaucoma.

Patients were followed up for at least 12 months. Gender, age, diagnosis, disease stage, etiology, and number of visits were collected. Treatments administered at Peking University Third Hospital were recorded, including conservative treatments (types and numbers of antiglaucoma medications) and surgical interventions (types and numbers of surgeries). Medical costs were defined as the total expenses incurred during the patient's healthcare encounters at our hospital, including both outpatient and surgical costs. Patients were categorized into 4 age strata for cost comparison: under 18 years, 18–45 years, 45–75 years, and over 75 years. Grouping was determined based on the etiological characteristics of NVG. In minors (under 18 years), the main causes of NVG are Coats disease and ocular tumors, rather than diabetes or retinal vein occlusion (RVO) as seen in adults (17). In patients over 45 years, the incidence of diabetes increases significantly and serves as a cutoff for diabetes-related eye disease (18). In those aged over 75 years, RVO becomes the predominant cause (17).

This study was conducted in accordance with the Declaration of Helsinki. All patients provided written informed consent, and the protocol was approved by the Ethics Committee of Peking University Third Hospital (Approval No. IRB00006761-2011199).

The statistical analysis was performed using SPSS 28.0. Normally distributed data were expressed as mean ± standard deviation, while non-normally distributed data were expressed as median with interquartile range (IQR). Quantitative data were analyzed using the independent-samples *t*-test or one-way ANOVA. *Post-hoc* multiple comparisons were conducted using the LSD method. A two-tailed *P*-value < 0.05 was considered statistically significant.

## 3 Results

### 3.1 Demographic characteristics of NVG patients

**Figure 1:**
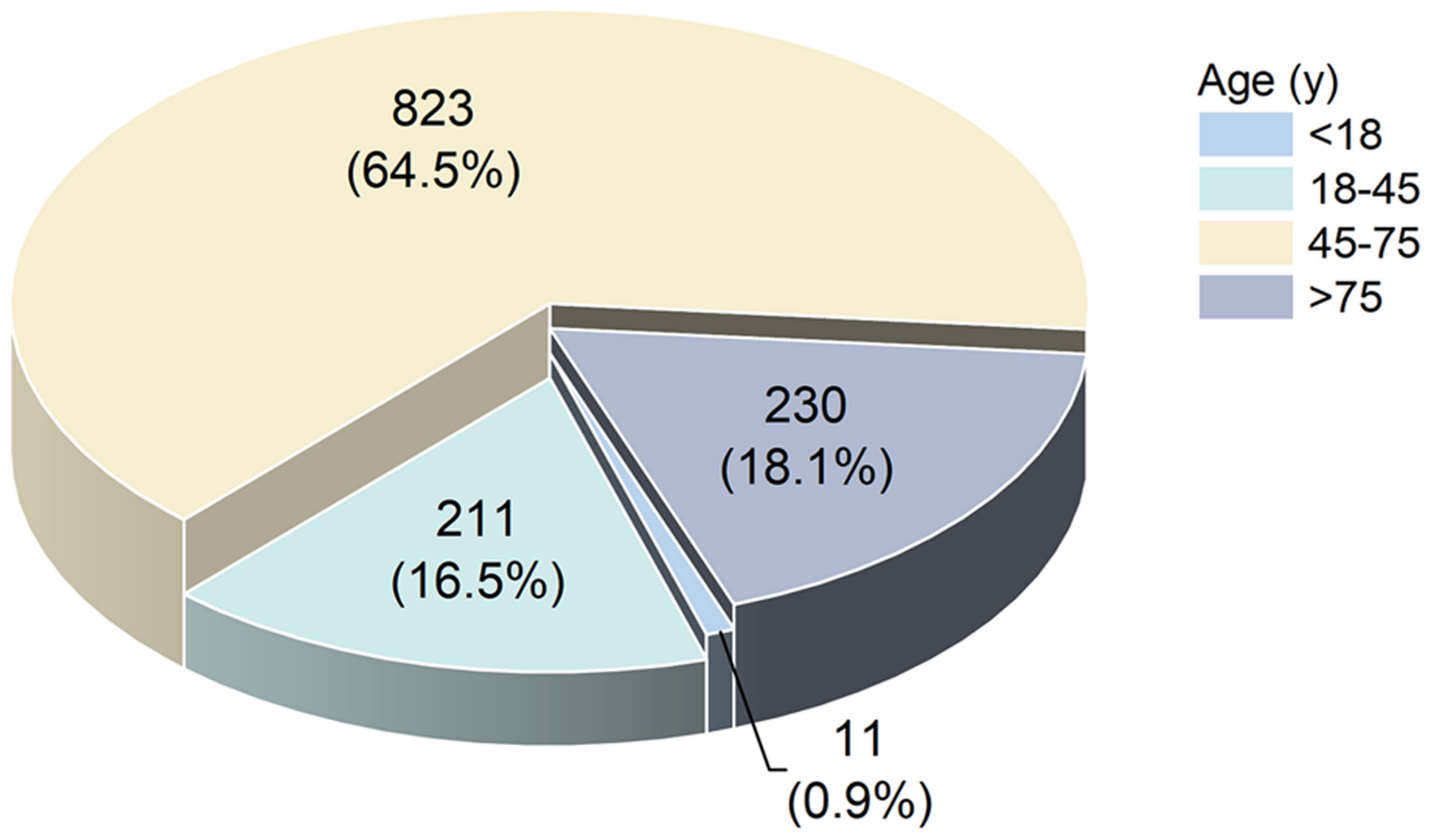
Number of neovascular glaucoma patients.

A total of 1275 patients were enrolled, representing 0.12% (1275/1091617) of all patients who visited the ophthalmology department during the study period (January 2016 to December 2024). Among these, there were 828 males and 447 females, with a male-to-female ratio of 1.9:1. The mean age at first visit was 59.9 ± 16.3 years (range 2–98), and males presented at a younger age than females (56.6 ± 16.1 years for male vs. 62.3 ± 14.8 years for female; *t* = −9.954, *p* < 0.001). 11 patients (0.9%) were under 18 years, 211 (16.5%) were aged 18–45 years, 823 (64.5%) were aged 45–75 years and 230 (18.1%) were over 75 years (Figure 1).

**Figure 2:**
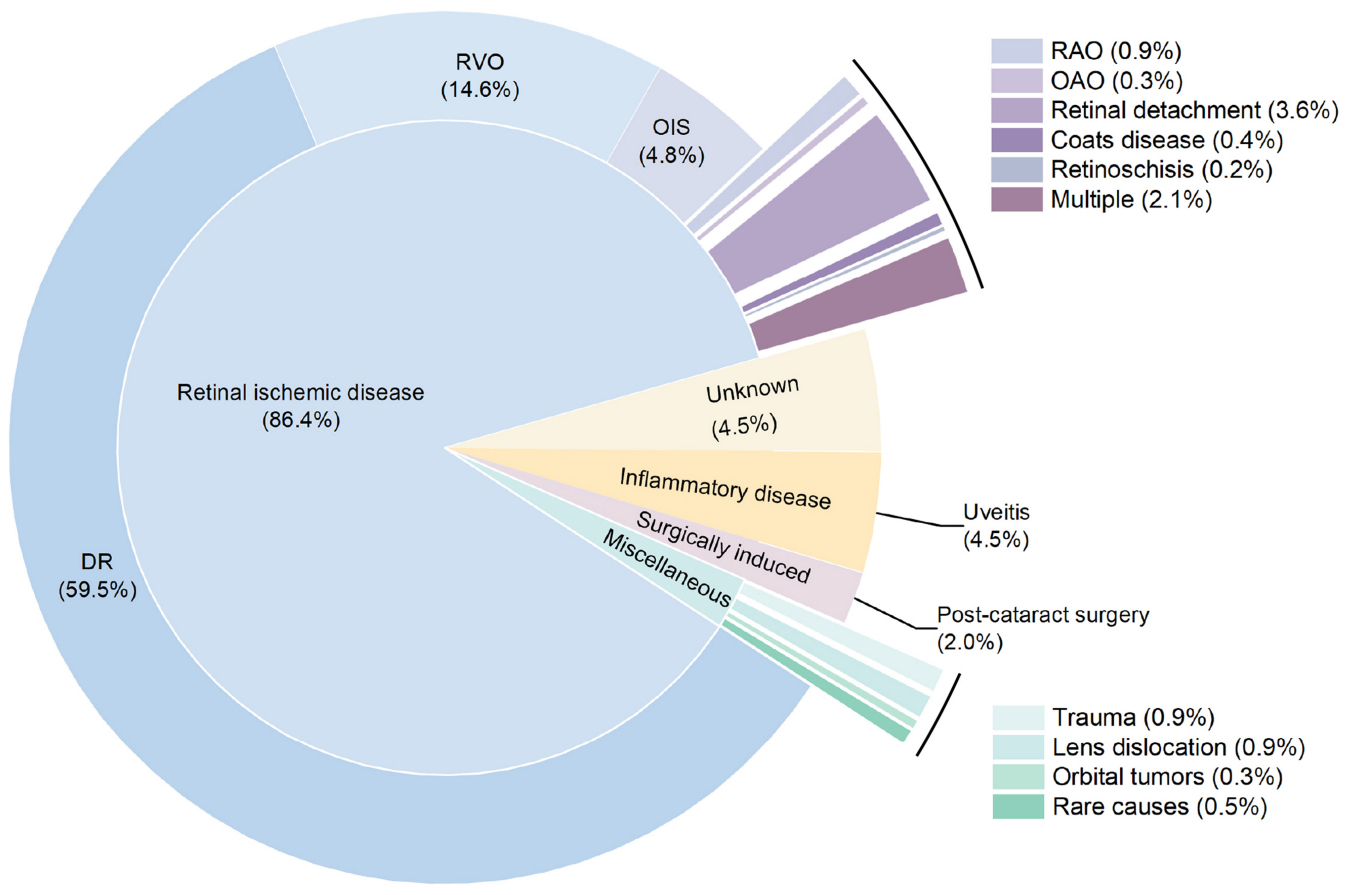
Etiology of neovascular glaucoma patients.

Six patients (0.5%) were diagnosed with iris neovascularization without glaucoma at the first visit, and the remaining 1,269 patients (99.5%) were diagnosed with NVG. The predominant cause of NVG was DR in 758 cases, followed by RVO in 186 cases, retinal artery occlusion (RAO) in 11 cases, ocular artery occlusion (OAO) in 4 cases, ocular ischemic syndrome (OIS) in 61 cases, uveitis in 57 cases, retinal detachment in 47 cases, Coats disease in 6 cases, retinoschisis in 2 cases, post-cataract surgery in 25 cases, trauma in 11 cases, lens dislocation in 11 cases, orbital tumors in 4 cases, rare causes in 7 cases (familial exudative vitreoretinopathy, persistent fetal vasculature, Takayasu's arteritis, ocular amyloidosis, retinal vasculitis, polycythemia vera, and leukocytosis-−1 case each), multiple etiologies in 27 cases (RVO and DR in 20 cases, CRAO and DR in 5 cases, CRAO and CRVO in 1 case, DR and OIS in 1 case), and unknown etiology in 58 cases (Figure 2; Table 1).

**Table 1:** Etiologies of NVG patients.

Causes of NVG	Number of cases
DR	758
RVO	186
RAO	11
OAO	4
OIS	61
Uveitis	57
RD	47
Coats disease	6
Retinoschisis	2
Post-cataract surgery	25
Trauma	11
Lens dislocation	11
Orbital tumor	4
Rare causes^a^	7
Multiple^b^	27
Unknown	58

NVG, neovascular glaucoma; DR, diabetic retinopathy; RVO, retinal vein occlusion; RAO, retinal artery occlusion; OAO, ophthalmic artery occlusion; OIS, ocular ischemic syndrome; RD, retinal detachment.

^a^Including familial exudative vitreoretinopathy (1 case), persistent fetal vasculature (1 case), Takayasu's arteritis (1 case), ocular amyloidosis (1 case), retinal vasculitis (1 case), polycythemia vera (1 case), and leukocytosis (1 case).

^b^Including RVO + DR (20 cases), CRAO + DR (5 cases), CRAO + CRVO (1 case), DR + OIS (1 case).

Treatment included medication, laser, and surgery. There were treatment records for 1,068 cases (83.8%). 611 patients (57.2%) received conservative treatment, among whom 450 (73.6%) required laser treatment subsequently. 457 patients (42.8%) received surgical interventions.

**Figure 3:**
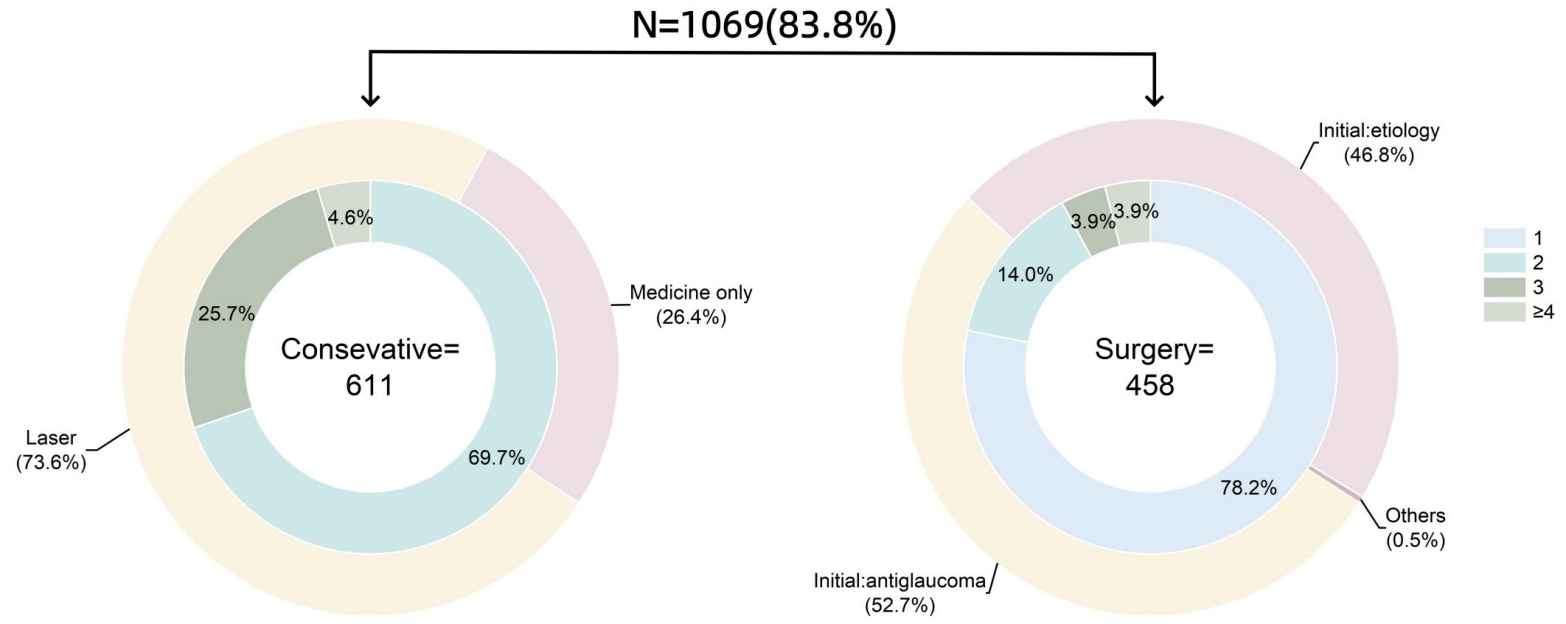
Treatment approaches for neovascular glaucoma patients.

The 611 conservatively treated cases used a mean of 2.4 ± 0.4 (range 2–6) antiglaucoma medications. 426 patients (69.7%) used 2 antiglaucoma medications, 157 (25.7%) used 3 antiglaucoma medications, and 28 (4.6%) used 4 or more medications (Figure 3).

Systemic antiglaucoma therapy (309 cases) consisted of 20% mannitol intravenous infusion and oral acetazolamide. 266 patients (86.1%) received acetazolamide alone, 12 patients (3.9%) received 20% mannitol infusion alone, and 31 patients (10.0%) received with a combination of both agents.

For topical medication, beta-blockers included carteolol hydrochloride eye drops (Mikelan, 430 administrations), timolol maleate eye drops (7 administrations), levobunolol hydrochloride eye drops (Betagan, 36 administrations), and betaxolol hydrochloride eye drops (Betoptic, 2 administrations). Alpha-agonists included brimonidine tartrate (Alphagan, 50 administrations). Carbonic anhydrase inhibitors included brinzolamide (Azopt, 467 administrations).

Among the surgical patients, 357 (78.2%) underwent 1 surgery, 64 (14.0%) underwent 2, 18 (3.9%) underwent 3, and 18 (3.9%) underwent more than 4 surgeries (Figure 3). Surgical interventions included vitreoretinal surgeries (intravitreal injection, vitrectomy combined with PRP, silicone oil tamponade, and silicone oil removal) and antiglaucoma surgeries (trabeculectomy, GDI, CPC, cyclocryotherapy) and enucleation. Initial surgery for the primary cause in 214 cases (46.8%), including 175 cases of anti-VEGF injection and 39 cases of vitrectomy. Initial surgery for antiglaucoma was performed in 241 cases (52.7%), including 177 cases of CPC, 51 cases of trabeculectomy, and 14 cases of GDI. 2 cases (0.5%) received enucleation.

### 3.2 Clinical profiles of NVG patients

The treatment duration in our hospital ranged from 1 to 2822 days, with a median of 49.8 days (IQR 10.7–190.0). 918 patients (72.0%) had a treatment duration of less than 30 days, 269 patients (21.1%) between 30 days and 1 year, and 88 patients (6.9%) longer than 1 year.

**Figure 4:**
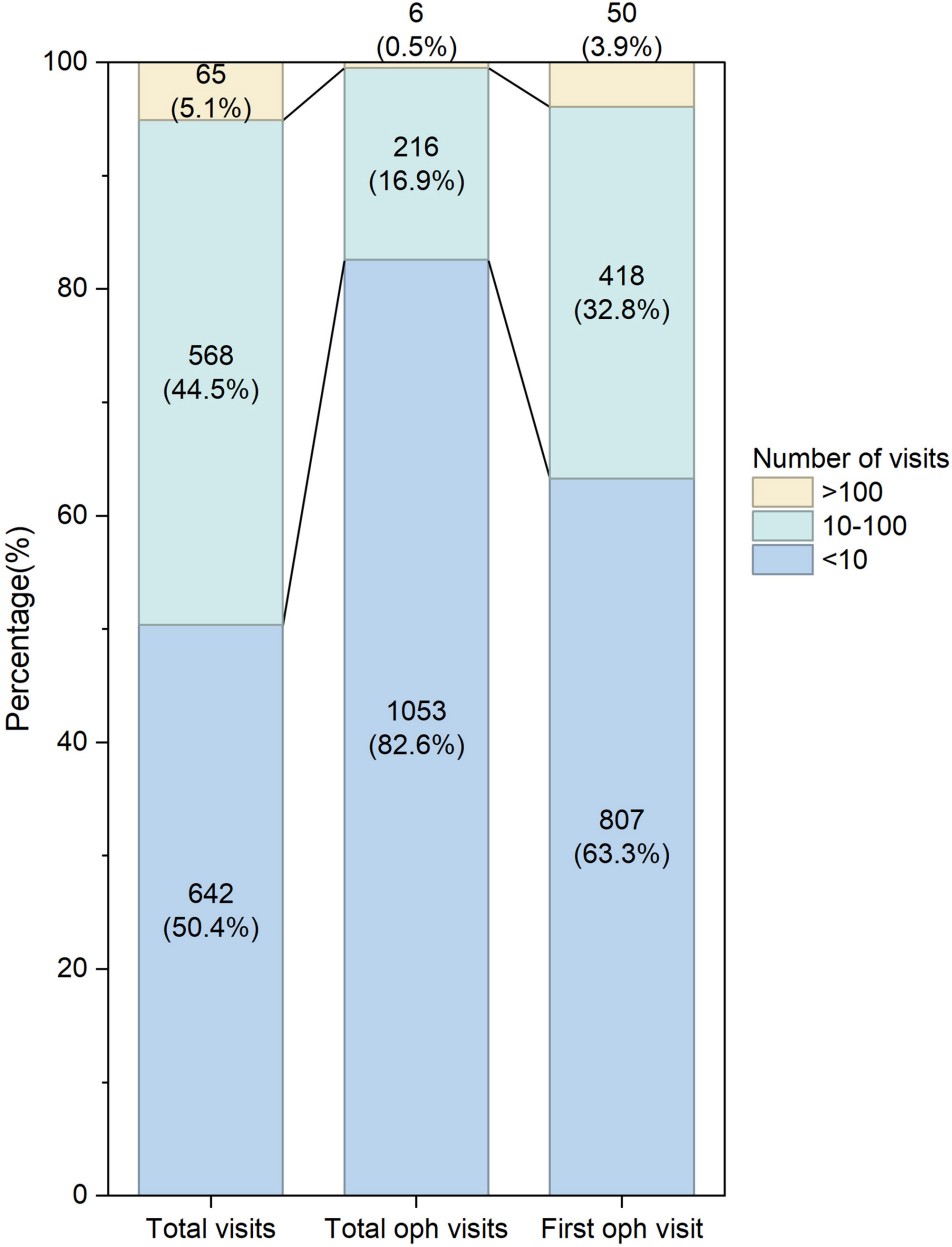
Clinical profile of neovascular glaucoma patients.

The number of ophthalmology department visits per patient ranged from 1 to 210, with a median of 2 visits (IQR 1–7). 1,053 patients (82.5%) had 1–10 ophthalmic visits, 216 patients (16.9%) had 11–100 visits, and 6 patients (0.5%) had more than 100 visits. The number of visits per patient at Peking University Third Hospital ranged from 1 to 703, with a median of 8 (IQR 3–24). 642 patients (50.4%) attended 1–10 visits, 568 patients (44.5%) attended 11–100 visits, and 65 patients (5.1%) attended more than 100 visits. The majority of patients presented initially to the ophthalmology department, though some with multiple systemic conditions had prior visits to other departments. The first visit to the ophthalmology department occurred at the 1st−703rd visit in our hospital, with a median of 4 (IQR 2–17). A total of 807 patients (63.3%) had their first ophthalmology consultation within visits 1–10, 418 patients (32.8%) within visits 11–100, and 50 patients (3.9%) beyond 100 visits (Figure 4).

Patients had a cumulative total of 3,343 visits, with an average cost per visit of 399.8 ± 383.4 yuan (range 60–4,825). The average cost was 399.9 ± 380.9 yuan (range 60–3,582.48) for males and 399.4 ± 388.4 yuan (range 60–4,825) for females, showing no statistically significant difference (*t* = 0.41, *p* = 0.967). Analysis by age group showed that the under 18 age group had 368.3 ± 365.3 yuan (range 60–1,345.92) per visit, 18–45 age group had 365.1 ± 352.3 yuan (range 60–2,771.6), 45–75 age group had 400.1 ± 376.1 yuan (range 60–3,582.5), and over 75 age group had 399.8 ± 383.4 yuan (range 60–4,825.0). The 45–75 age group had the highest average cost per visit, and the difference was statistically significant (*F* = 3.615, *p* = 0.0128). LSD *post-hoc* tests indicated significant differences between the 45–75 age group and the 18–45 age group (*p* = 0.042) as well as the over 75 age group (*p* = 0.033); a significant difference was also found between the 18–45 age group and the over 75 age group (*p* = 0.033).

The average surgical cost was 8334.1 ± 10019.0 yuan (range 2,000–50,000), with male patients averaging 8,056.5 ± 9,689.2 yuan (range 2,000–50,000), while female patients averaged 8,856.3 ± 1,0623.2 yuan (range 2,000–47,000), showing no statistically significant difference between the two groups (*t* = −0.816, *p* = 0.415). Surgical costs across different age groups were as follows: under 18 age group 5,000.0 ± 4,761.0 yuan (range 2,000.0–12,000.0); 18–45 age group 9,379.3 ± 10,543.6 yuan (range 2000–40000); 45–75 age group 8,833.3 ± 10,471.6 yuan (range 2,000–50,000); over 75 age group 4,428.6 ± 5,204.6 yuan (range 2,000–48,000). There was a statistically significant difference in surgical costs among the groups (*F* = 3.954, *p* = 0.008). *Post-hoc* multiple comparisons using LSD showed that the over 75 age group had the lowest surgical costs, with statistically significant differences compared to the 18–45 age group and the 45–75 age group (*p* = 0.003 and 0.002, respectively).

## 4 Discussion

This study analyzed the medical visit profiles for NVG patients in a tertiary medical center in China from 2016 to 2024. The findings suggested that the onset age of NVG peaked between 45–75 years, and was more common in males, who also tended to present at a younger age than females. At the initial visit, 6 patients (0.5%) were diagnosed with iris neovascularization without glaucoma, while the remaining 1269 patients (99.5%) were diagnosed with NVG. The predominant cause of NVG was DR, followed by retinal vascular occlusion and other rare etiologies.

Over a 9-year period, 82.5% of patients had 10 or fewer visits (including 10 visits), and 72% had an encounter span of 30 days or less. Most patients presented first to ophthalmology, though some with multiple systemic conditions had prior visits to other departments. The median cumulative visit number at the first ophthalmology consultation was 4. 475 cases (53.0%) had their first ophthalmology consultation by their 5th visit to our hospital.

Lin et al. and Abdullah et al. reported NVG was more common in males, with a male-to-female ratio of approximately 2:1 (17, 19), which was consistent with our study. It is possibly due to a higher prevalence for males of underlying diseases such as DR and retinal vascular occlusion (20). We also propose that higher rates of smoking and alcohol consumption, along with poorer treatment adherence among males may promote progression of underlying ischemic retinal diseases toward NVG (21, 22). Conversely, the protective effect of estrogen in glaucoma may also apply to NVG (23–25). Studies have indicated that postmenopausal women showed increased glaucoma incidence, while estrogen therapy may delay onset (23). Estrogen can protect retinal ganglion cells from damage under high intraocular pressure (IOP). Potential mechanisms include promoting axon regeneration, anti-inflammatory and anti-apoptotic effects, and improved local perfusion (24, 25). Estrogen can inhibit retinal ganglion cell loss, prevent tau cleavage in optic nerve head astrocytes, and thereby decrease neurofibrillary tangles (25–27). In an oxygen-induced retinopathy (OIR) model, estrogen receptor β agonists downregulate VEGF and suppress pathological neovascularization, suggesting a possible role in NVG (28). Additionally, estrogen may promote IOP homeostasis by affecting extracellular matrix turnover, focal adhesion assembly, actin stress fiber formation, mechanosensation, and nitric oxide production to protect against glaucoma (29).

NVG primarily affects middle-aged and older individuals, most commonly between 45 and 75 years of age. Studies have indicated a rise in the prevalence of diabetes after the age of 45 (30), and the prevalence of hypertension also increases with age (31). Given the earlier onset of these systemic conditions, along with increased life expectancy, this trend likely contributes to making 45–75 years the high-risk period for NVG. DR is a major cause of NVG. There is a prioritized community-based screening, health education, and digital informationization for DR in China. The cost-effectiveness of digital technologies for age-related eye diseases has been demonstrated (13), and a novel full-process digital-empowered hierarchical vision management strategy has proven cost-effective for visual impairment (32). These approaches enable stratified healthcare management according to age and gender, potentially reducing the economic burden of NVG.

Treatments of NVG currently include PRP, antiglaucoma surgery, and vitrectomy (3, 4). In this study, 42.8% of patients underwent surgical treatment. Most (78.2%) underwent 1 surgery; 18 cases (3.9%) underwent 4 or more surgeries. Initial treatment was equally divided between etiology (46.8%) and antiglaucoma surgery (52.7%). Surgical management of NVG involves various approaches. For visual function preservation, triple sequential therapy has been shown to be more effective than CPC in our previous research (5). Similarly, Lin et al. found that both trabeculectomy with mitomycin C combined with intravitreal anti-VEGF injection (IVAV) and Ahmed glaucoma valve (AGV) surgery combined with IVAV achieved better IOP reduction at 6 months compared to CPC (7). Other studies have reported that AGV and CPC had comparable IOP reduction at 6 months, but the latter was associated with higher failure rates, reoperation rates, and worse visual outcomes (33). In terms of improving the surgical success rate and the reducing reoperation rate, Baerveldt glaucoma implant (BGI) was more effective than AGV (34). Trabeculectomy and BGI showed no significant difference in IOP control at 12 months, but trabeculectomy was associated with fewer late complications (35), which partly explains why this procedure was more frequently adopted in this study. Additionally, for patients with clear optical media, anti-VEGF monotherapy may be a feasible option (36). In summary, various surgical approaches were utilized in this study depending on individual conditions. The rates of initial treatment for the underlying disease vs. glaucoma were comparable (46.8% vs. 52.7%), reflecting a patient-tailored approach based on whether managing the underlying cause or controlling IOP was clinically prioritized.

Earlier studies have focused on the cost of treatment for open-angle glaucoma (14, 37, 38), but none have addressed the number of visits and medical costs associated with NVG. This study was based on 9 years of single-center cohort data to analyze patient demographics, disease profiles, and treatment methods. Additionally, it documented the number of patient visits, treatment duration, and medical costs.

This study is the first to describe the disease profile and medical costs of NVG patients in China, finding a gender difference and variations in medical costs among different age groups. Patients had a total of 3,343 visits. Analysis of visit costs revealed that individuals aged 45–75 years had the highest outpatient cost per visit. This may be due to their greater need for visual function preservation and a higher likelihood of systemic comorbidities, leading to more preoperative examinations and thus higher medical expenses. The average cost per surgery was approximately 8,334.1 yuan. Surgical costs were highest among the 18–45 age group, likely because this group has the strongest demand for preserving visual function, and earlier disease onset often indicates greater severity and more frequent surgical interventions, resulting in higher overall surgical costs. The over-75 age group had the lowest cost, which may be related to poor tolerance and unfavorable prognosis in patients of this age group. Therefore, surgical strategies tend to be more conservative, focusing on symptom relief and choosing relatively simple procedures.

However, this study has several limitations. First, the data were derived from a single high-level medical center, which may not reflect the actual conditions or economic burden in lower-level or rural areas. Future research should therefore conduct multi-regional, multi-center studies. Second, the study mainly analyzed the costs over the treatment duration. However, NVG is a chronic disease that demands lifelong management, and thus treatment costs continue beyond the study period. Long-term follow-up is needed to evaluate the economic burden. Third, the cost analysis was limited to descriptive and univariate methods, without adjusting for confounders such as gender, etiology, and clinical stage. Given that treatment costs of glaucoma are associated with disease severity (14), and NVG is a refractory glaucoma, costs may rise substantially with progression. Future studies should use multivariable regression to assess the independent impact of clinical stage and patient factors on costs. Fourth, only direct medical costs (inpatient and surgical costs) were considered. Indirect costs arising from unemployment, travel expenses, and caregiver burden would impose a higher real-world socioeconomic burden (39).

## 5 Conclusions

This study found that NVG in China predominantly affects males, with the age of onset concentrated between 45 and 75 years. The most common cause was DR. The overall medical costs were highest among patients aged 45–75 years. Outpatient visit expenses per episode peaked in the 45–75 age group, while surgical costs were highest in the 18–45 age group.

## Data Availability

The data analyzed in this study is subject to the following licenses/restrictions: The data contain confidential patient health information from the Hospital Information System (HIS). Access is restricted by the Ethics Committee of Peking University Third Hospital. The data are not publicly available. Requests to access these datasets should be directed to Ying Hong, drhongying@bjmu.edu.cn.
